# Interpretable CT-Based Radiomics for Prediction of High-Affinity Neoantigens Associated Immunogenic States in Advanced Hepatocellular Carcinoma Treated with TACE Plus Targeted Therapy and Immunotherapy

**DOI:** 10.3390/cancers18193218

**Published:** 2026-10-06

**Authors:** Chaofan Bian, Yanjin Qin, Hongliang Zou, Wenzhe Fan, Jiaping Li

**Affiliations:** 1 Department of Interventional Oncology, The First Affiliated Hospital of Sun Yat-Sen University, Guangzhou 510080, China; bianchf@mail2.sysu.edu.cn (C.B.);; 2 Department of Radiology, The First Affiliated Hospital of Sun Yat-Sen University, Guangzhou 510080, China; 3 Affiliated Cancer Hospital and Institute of Guangzhou Medical University, Guangzhou 510095, China

**Keywords:** hepatocellular carcinoma, CT radiomics, tumor immunogenicity, TACE

## Abstract

**Objectives:** To develop and validate a CT radiomics-based machine learning model for the noninvasive prediction of HANS status in advanced HCC and to investigate the prognostic and genomic characteristics associated with HANS status. **Materials and Methods:** This retrospective study included patients with advanced HCC treated with TACE-based triplet therapy. Patients were followed until death or last follow-up. Tumor tissue underwent whole-exome sequencing for HANS calculation, and arterial-phase CT images were used for radiomic feature extraction. A radiomics model (HANS-Rad), a clinical model (HANS-Clin), and a combined model (HANS-RC) were developed using least absolute shrinkage and selection operator regression. Model performance was evaluated using receiver operating characteristic curve analysis. Calibration and decision curve analyses were performed to assess model reliability and clinical utility. Shapley Additive Explanations (SHAP) were used for model interpretation. Genomic analyses were conducted to explore differences between predicted HANS subgroups. **Results:** A total of 129 patients were included (median age, 54 years; IQR, 44–62 years; 90% men). The HANS-High group demonstrated longer overall survival compared with the HANS-Low group (median, 23.0 vs. 10.0 months; hazard ratio, 0.60; *p* = 0.026). The combined HANS-RC model achieved the best performance, with an area under the curve (AUC) of 0.964 in the training set and 0.869 (95% CI, 0.716–1.000) in the test set, outperforming the radiomics-only and clinical models. The model also showed favorable calibration and net benefit. Genomic analyses revealed that HANS-High tumors exhibited higher tumor mutational burden and enrichment of immune-related and oncogenic pathways, whereas HANS-Low tumors were associated with distinct mutational patterns and pathway alterations. **Conclusions:** A radiomics-based machine learning model using CT images enables the noninvasive prediction of HANS status in advanced HCC, with strong prognostic relevance and biologic interpretability supported by genomic analyses.

## Simple Summary

Patients with advanced hepatocellular carcinoma can respond differently to combination treatments that include transarterial chemoembolization, targeted therapy, and immunotherapy. Tumor immunogenicity, which reflects how strongly a tumor may be recognized and attacked by the immune system, may influence treatment response and long-term outcomes. However, assessing tumor immunogenicity usually requires tumor tissue and genomic testing, which may not be feasible for all patients. In this study, we investigated whether routinely obtained computed tomography images could provide a non-invasive estimate of tumor immunogenicity. Using data from 129 patients with advanced hepatocellular carcinoma, we developed an interpretable imaging-based model to predict high-affinity neoantigen-associated immunogenic states. The imaging-based predictions were also associated with genomic alterations, tumor mutation burden, immune-related pathways, and clinical outcomes. Our findings suggest that computed tomography radiomics may provide a non-invasive approach for estimating tumor immunogenicity and may support treatment stratification and imaging-based precision oncology.

## 1. Introduction

Hepatocellular carcinoma (HCC) accounts for 85–90% of primary liver malignancies [1]. In China, its incidence and mortality rank fifth and second among all cancers [2], respectively. Chronic HBV infection drives over 80% of HCC cases, typically following a hepatitis–cirrhosis–cancer sequence. Curative treatments (resection, ablation, transplantation) yield 5-year survival >50%, but 70–80% of patients present with unresectable disease due to multifocality, large burden, vascular invasion, or poor liver reserve [2,3].

The triple regimen of transarterial chemoembolization (TACE), immune checkpoint inhibitor (ICI), and anti-angiogenic agent is now first-line for unresectable intermediate/advanced HCC [4,5,6]. Three Phase 3 trials (EMERALD-1, LEAP-012, TALENTACE) showed superiority over TACE alone [7,8]. However, as noted in GUIDANCE001, the optimal population for triple therapy is undefined, and overtreatment increases toxicity [9]. The limitation stems from current staging systems (TNM, BCLC) relying on gross morphology [10], whereas HCC is molecularly heterogeneous. The tumor immune microenvironment (TIME) varies widely [11], even spatially within the same tumor, dictating immunotherapy response [6,12,13]. Traditional staging cannot guide patient selection.

The dynamic interplay between tumor and host immunity governs long-term disease control and the efficacy of combination immunotherapy [12,14,15]. We previously reported in the gut a high-affinity neoantigen score (HANS)—the number of neoantigens with mutant-class I MHC binding IC_50_ < 50 nM. In 56 HCC patients, HANS correlated with overall survival, while conventionally, the survival discriminative ability of TMB is limited [16]. HANS strongly associated with tumor-infiltrating CD39^+^CD8^+^ T cells; HANS-specific T cells exhibited antitumor activity in organoid assays. HANS-High patients had more effector-phenotype CD39^+^PD-1_int_CD8^+^ TILs and better response to anti-PD-1 therapy. However, those findings came from a treatment-naive mixed cohort; the HANS value in patients with unresectable advanced HCC receiving TACE plus targeted agents and ICIs is unknown.

Radiomics enables the high-throughput extraction of subtle texture and heterogeneity features from conventional medical images, bridging macroscopic phenotypes and microscopic molecular events [17,18,19]. Evidence indicates that radiomic features are associated with the HCC immune microenvironment, suggesting that the noninvasive dissection of tumor immune characteristics is feasible [20,21,22].

In this study, we first validated HANS as a prognostic stratifier for triple therapy (TACE-based triplet therapy) using whole-exome sequencing, then built a CT radiomics-based model to predict HANS noninvasively, and elucidated the model’s genomic basis with multi-omics data. Our goal is to provide an imaging-based tool to noninvasively predict HANS-associated immunogenic states and support prognostic stratification.

## 2. Materials and Methods

### 2.1. Study Design and Population

**Figure 1:**
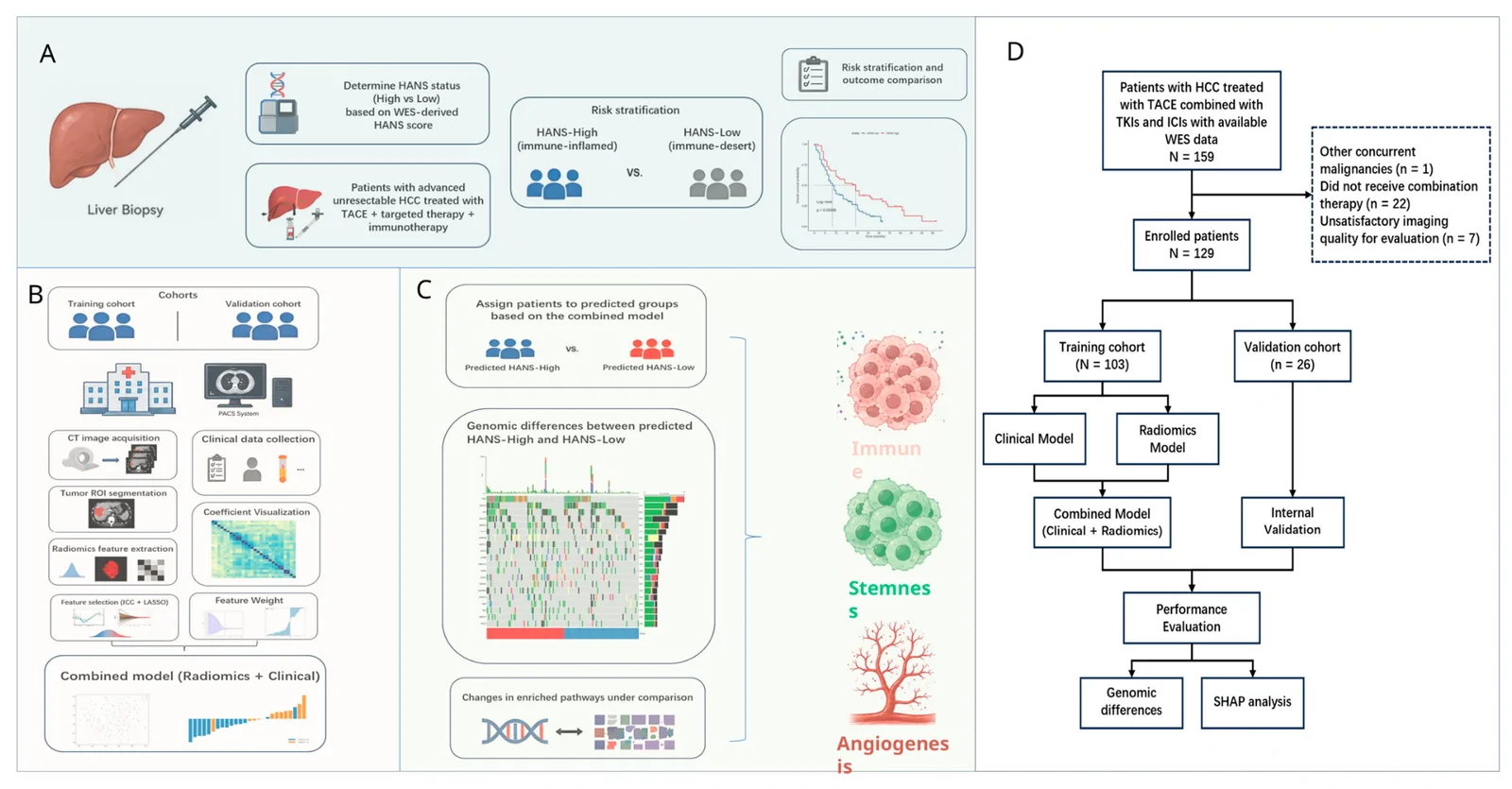
(**A**) WES-derived HANS stratified patients with unresectable HCC treated with TACE plus targeted therapy and immunotherapy into HANS-High and HANS-Low groups, with survival compared. (**B**) A radiomics–clinical model was built using CT and clinical data, including tumor segmentation, feature extraction, and selection (ICC, LASSO). (**C**) Model-based stratification enabled the comparison of genomic alterations and pathway enrichment, highlighting differences in immune activity, stemness, and angiogenesis. (**D**) Workflow of patient selection, cohort division, model construction, validation, and downstream analyses including genomic comparison and SHAP interpretation.

Patients with HCC who underwent TACE combined with antiangiogenic and ICIs were recruited between January 2015 and December 2023. Inclusion criteria were (1) age 18–75 years; (2) Barcelona Clinic Liver Cancer (BCLC) stage B or C; (3) pathologically confirmed HCC with tumor tissue available for whole-exome sequencing (WES); (4) Child–Pugh class A or B; (5) prothrombin time < 20 s; (6) platelet count ≥ 50 × 10^9^/L; and (7) contrast-enhanced abdominal computed tomography (CT) performed within 2 weeks before treatment. Exclusion criteria were presence of severe medical comorbidities, history of other malignancies, and poor image quality (Figure 1).

This study was reviewed and approved by the Institutional Ethics Review Board of The First Affiliated Hospital, Sun Yat-sen University (IRB No. [2018] No. 278).

### 2.2. Pathological and Immunogenicity Assessment

All enrolled cases had pre-operative or baseline CT-guided biopsy specimens. Genomic DNA was extracted from tumor tissue and matched peripheral blood samples, followed by library construction and exome capture. Paired-end sequencing was performed on an Illumina high-throughput sequencing platform(Illumina, San Diego, CA, USA). Somatic mutations were identified using a standard bioinformatics pipeline, and only non-synonymous mutations were retained for subsequent analysis.

HLA-I typing (HLA-A, HLA-B, HLA-C) was performed based on WES data. The NetMHCpan algorithm was used to predict the binding affinity of mutant peptides to the patient’s own HLA molecules. Binding affinity was expressed as half-maximal inhibitory concentration (IC_50_, nM). According to previous studies, mutant peptides with IC_50_ < 50 nM were defined as high-affinity neoantigens. The number of high-affinity neoantigens per patient was designated as the HANS.

### 2.3. Treatment Protocol

The treatment regimen consisted of TACE combined with an immune checkpoint inhibitor (ICI) and antiangiogenic therapy. ICIs included PD-1/PD-L1 monoclonal antibodies, whereas antiangiogenic agents included the tyrosine kinase inhibitors sorafenib and lenvatinib. TACE is performed under digital subtraction angiography guidance with selective catheterization into the tumor-feeding artery, followed by an infusion of chemotherapeutic agents (epirubicin) and embolic materials (lipiodol or drug-eluting beads). The procedure is individualized based on tumor burden, vascular anatomy, and liver function, and can be repeated as needed. Immunotherapy is given intravenously at standard doses of PD-1/PD-L1 antibodies until disease progression or unacceptable toxicity. TKIs are taken orally, with starting doses determined by body surface area, liver function, and tolerability, and adjusted according to adverse events.

The enrolled patients were followed up every 4–12 weeks. Follow-up was set for 30 June 2025. Overall survival (OS) refers to the duration from the commencement of treatment until the occurrence of mortality from any cause.

### 2.4. CT Scanning Protocol and Image Acquisition

All patients underwent biphasic contrast-enhanced CT within two weeks before treatment, followed by monthly CT scans after treatment initiation. More details are shown in the Supplementary Materials.

### 2.5. Image Preprocessing and Tumor Segmentation

Arterial- and portal venous-phase DICOM images were exported from the picture archiving and communication system (PACS). However, only arterial-phase images were used for tumor segmentation and radiomic feature extraction; portal venous-phase images did not contribute any radiomic features to model development. DICOM pixel values were converted to Hounsfield units (HU) using the corresponding rescale slope and intercept. Two abdominal radiologists, each with at least 5 years of experience, manually performed slice-by-slice three-dimensional tumor segmentation on arterial-phase images using ITK-SNAP(4.0.1) while excluding visible vessels, bile ducts, and necrotic areas. The CT images and corresponding segmentation masks were subsequently resampled to an isotropic voxel size of 1.0 × 1.0 × 1.0 mm^3^. Voxel intensities were retained on the HU scale, without normalization to 0–255. Before radiomic texture feature calculation, voxel intensities within the segmented tumor were discretized using a fixed bin width of 25 HU. Intra- and interobserver reproducibility was evaluated using intraclass correlation coefficients (ICCs), and only features with both intra- and interobserver ICCs greater than 0.85 were retained.

### 2.6. Radiomic Analysis

The training-to-validation split ratio was 8:2. All three models were developed on the same training set (*N* = 103) and tested on the same independent test set (*N* = 26) using a fixed random seed (0). All radiomic features were first normalized using Z-score standardization. Feature stability was assessed using the intraclass correlation coefficient (ICC), and only highly reproducible features (ICC ≥ 0.85) were retained. Features associated with the study endpoint were then screened using the Mann–Whitney U test (*p* < 0.05). To reduce multicollinearity, pairwise Spearman correlation analysis was performed; for highly correlated feature pairs (|ρ| > 0.9), the feature with the stronger univariable association was retained. Finally, LASSO regression with 10-fold cross-validation was used for feature selection and overfitting reduction, with the optimal λ determined by the minimum cross-validated error.

### 2.7. Predictive Models Construction and Validation

Using HANS-High/Low group as the outcome variable, the selected radiomics features were then used to construct a radiomics model (HANS-Rad) by machine learning classifiers, including logistic regression (LR), support vector machine (SVM), multilayer perceptron (MLP), k-nearest neighbors (KNN), random forest (RF), extra trees, eXtreme Gradient Boosting (XGBoost), and Light Gradient Boosting Machine (LightGBM).

For the clinical model (HANS-Clin), 29 variables were included initially. After the same normalization and correlation filtering, 27 features underwent LASSO regression, which retained 13 features.

To build the combined model (HANS-RC), the four highest-weight clinical features from LASSO (sex, age, tumor number, and combined AFP/CA19-9 elevation) were merged with the 40 radiomic features, giving a total of 44 features. A second LASSO run selected 36 non-zero coefficients—all four clinical features plus 32 radiomic features—and these were used to construct the combined model (Figure S1). Model training was performed in the training cohort, and hyperparameters were optimized using grid search with cross-validation. During model selection, 11 classifiers were systematically compared across the clinical, radiomic, and combined models. Finally, the MLP classifier was selected as the optimal model based on its performance. Model performance was evaluated using the AUC, Harrell’s concordance index (C-index), calibration curves, Brier score, and decision curve analysis (DCA).

### 2.8. Statistical Analysis

Continuous variables were expressed as mean ± standard deviation or median (interquartile range), depending on their distribution, and categorical variables as frequencies and percentages. Between-group comparisons were performed using Welch’s *t*-test or the Mann–Whitney U test for continuous variables, and the chi-square test or Fisher’s exact test for categorical variables, as appropriate. Radiomic modeling and statistical analyses were primarily performed using Python 3.8.6. Radiomic features were extracted using PyRadiomics 3.1.0. The recorded software environment for the additional validation analyses included scikit-learn 1.3.2, NumPy 1.24.4, and pandas 2.0.3. The original XGBoost candidate models were generated using XGBoost 1.5.2, and Matplotlib 3.2.2 was used to generate the original model-related figures. Neoantigen binding affinity was predicted using NetMHCpan 4.1, and genomic analyses were performed using R 4.5.1. Additional figures were generated using GraphPad Prism 9 or R, as appropriate. Model performance was assessed using the area under the receiver operating characteristic curve (AUC), accuracy, sensitivity, specificity, calibration, Brier score, and decision curve analysis. All statistical tests were two-sided, and a *p* value < 0.05 was considered statistically significant.

## 3. Results

### 3.1. Baseline Characteristics and HANS Status Distribution

A total of 129 patients with advanced hepatocellular carcinoma (HCC) treated with TACE-based triplet therapy were included. In the overall cohort, the majority were male (90%), median age was 54 years (IQR 44–62), hepatitis B virus (HBV) infection rate was 90%, 80% were BCLC stage C, 75% were CNLC stage 3, 77% had Child–Pugh class A, and 64% had liver cirrhosis. Regarding tumor burden, 72% had multiple tumors (>3 nodules), 83% had the largest diameter > 5 cm, portal vein tumor thrombus (PVTT) was present in 68%, and extrahepatic metastasis in 37%. HANS values were calculated from whole-exome sequencing data of tumor tissue. Using the median HANS as the cutoff, 70 patients (54.3%) were assigned to the HANS-Low group and 59 (45.7%) to the HANS-High group.

The HANS-High group had a higher proportion of patients > 50 years. The HANS-Low group had more patients with multiple tumors (>3 nodules) (84% vs. 58%, *p* < 0.001) No significant differences were found in the remaining clinicopathological features, including BCLC/CNLC stage, sex, ECOG PS, Child–Pugh grade, cirrhosis, AFP level, tumor size, vascular invasion, or extrahepatic metastasis (Table 1).

Thus, they were well-balanced with respect to the key staging systems (BCLC and CNLC).

### 3.2. Association Between HANS Status and Patient Survival

**Figure 2:**
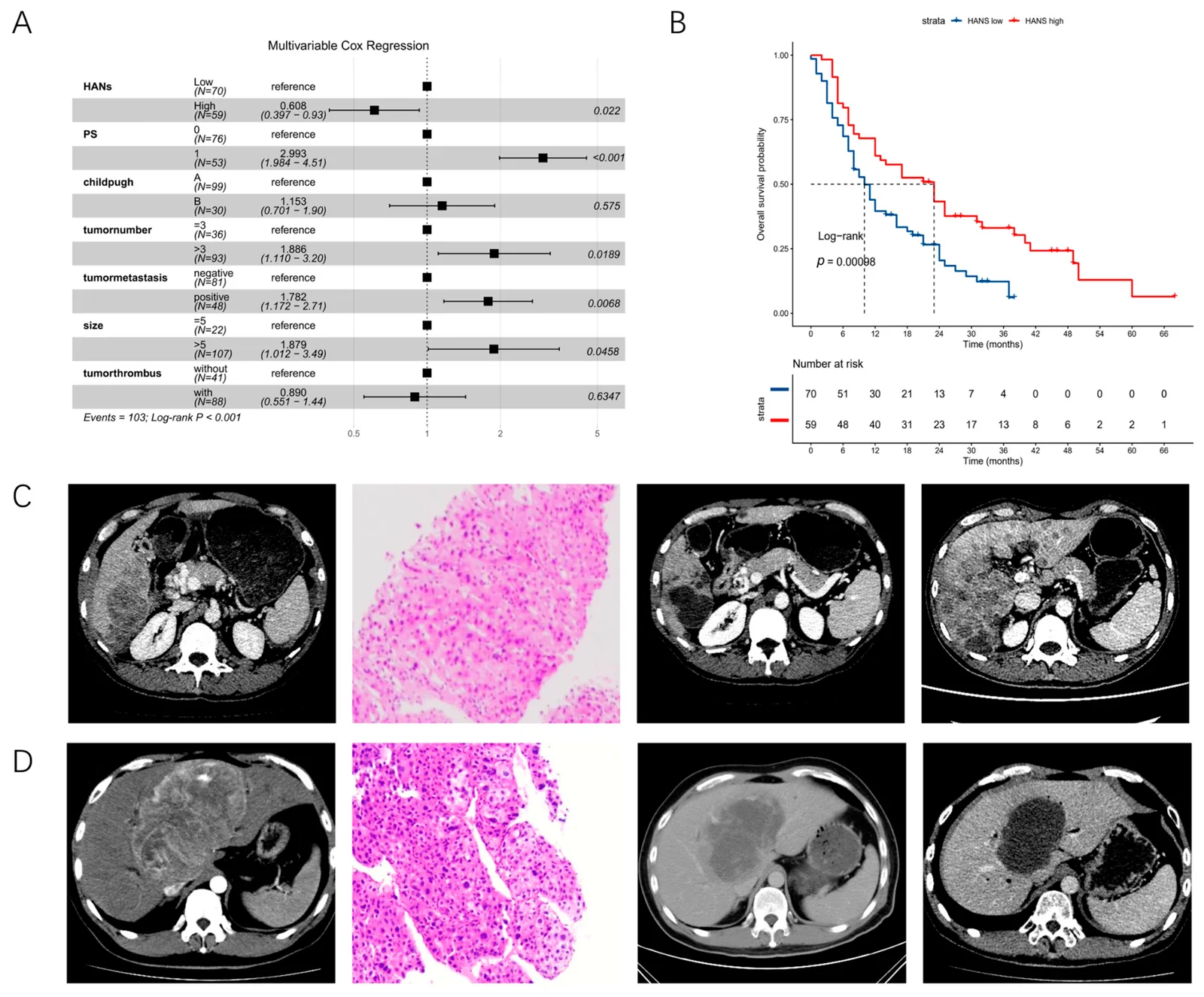
Survival analysis and representative imaging. (**A**) Multivariable Cox analysis including TMB, HANS, PS, Child–Pugh class, tumor number, metastasis, tumor size, and tumor thrombus; HANS-High remained independently associated with improved overall survival. (**B**) Kaplan–Meier curves comparing HANS-Low and HANS-High groups. (**C**,**D**) Representative cases showing baseline contrast-enhanced CT, corresponding H&E staining of biopsy specimens, and follow-up CT at 1 and 6 months after treatment.

At last follow-up, the median follow-up time was 38.0 months (95% CI 32.0–49.0, Table S1). Median overall survival (OS) was 10.0 months (95% CI 8.0–16.0) in the HANS-Low group and 23.0 months (95% CI 12.0–32.0) in the HANS-High group (Table S2). Multivariate Cox regression showed that high HANS was independently associated with longer OS (HR 0.60, 95% CI 0.39–0.95, *p* = 0.026, Figure 2A, Table S3 and Table S4).

Survival curves separated after 12 months, with higher OS rates in HANS-High at 12, 24, and 36 months (61.0% vs. 39.6%, 43.3% vs. 20.5%, and 33.1% vs. 12.3%, respectively).

As shown in the CT images of the patients, Case 1 (HANS-Low group) and Case 2 (HANS-High group) (Figure 2C,D).

### 3.3. Predictive Models Performance

**Figure 3:**
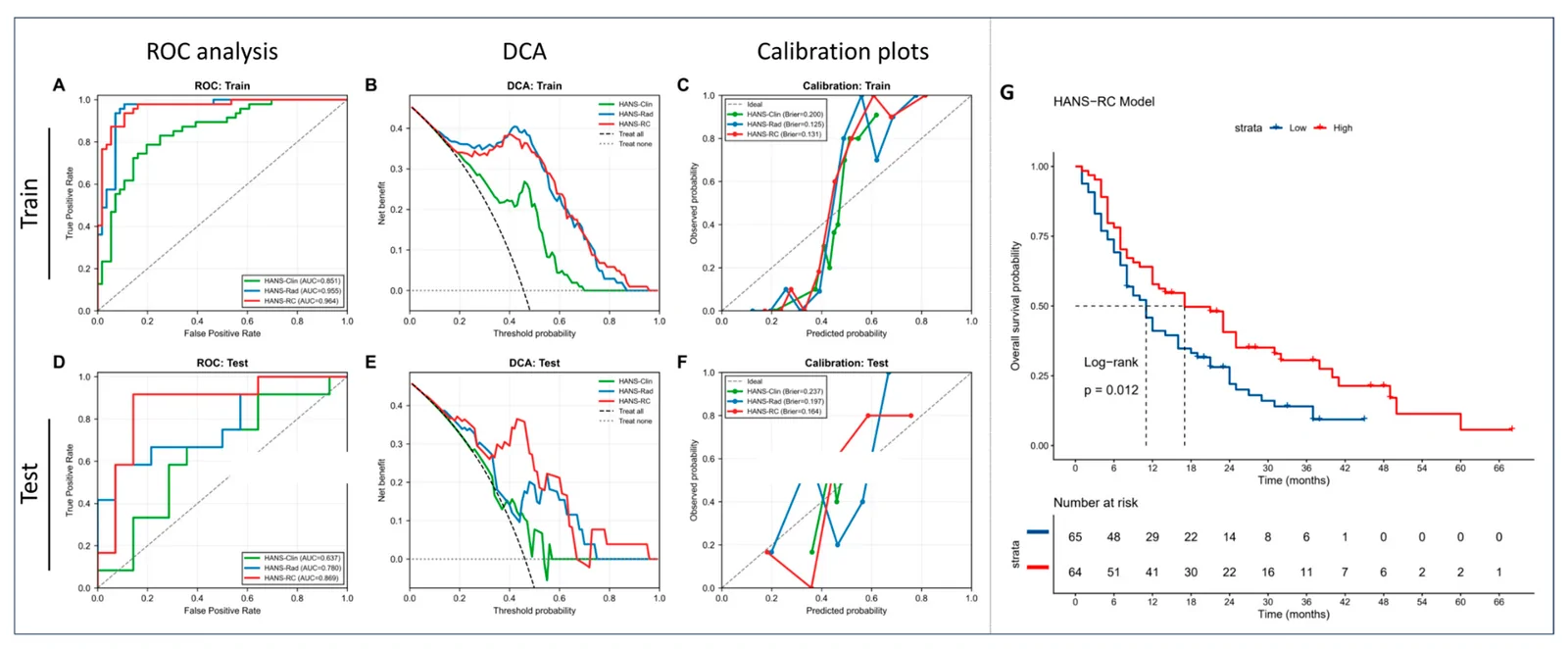
Model performance and clinical utility. (**A**,**D**) ROC curves in the training and test cohorts, demonstrating improved discrimination of the combined model (HANS-RC) over clinical (HANS-Clin) and radiomics-only (HANS-Rad) models. (**B**,**E**) Decision curve analysis showing higher net benefit of the combined model across a range of threshold probabilities. (**C**,**F**) Calibration plots indicating agreement between the predicted and observed probabilities, with lower Brier scores for the combined model suggesting better calibration. (**G**) Kaplan–Meier curves of overall survival in the combined (training and validation) cohort stratified by the HANS-RC model. Patients in the HANS-High group (red) showed significantly better survival than those in the HANS-Low group (blue) (log-rank *p* = 0.012). Tick marks indicate censored observations, and the numbers at risk at each time point are shown below the plot.

In the training cohort, the combined HANS-RC model showed the highest discriminative ability (AUC = 0.964), exceeding both the radiomics model (HANS-Rad, AUC = 0.955) and the clinical model (HANS-Clin, AUC = 0.851) (Figure 3).

In the test cohort, this ranking was maintained. HANS-RC achieved an AUC of 0.869 (95% CI 0.716–1.000), with an accuracy of 0.885, sensitivity of 0.917, and specificity of 0.857. In comparison, HANS-Rad yielded an AUC of 0.780 (95% CI 0.595–0.964), with higher specificity (0.929) but lower sensitivity (0.583). Although discrimination decreased from the training to the test cohort, the HANS-RC model retained good discriminatory performance in the test cohort (AUC = 0.869, 95% CI: 0.716–1.000). Calibration analysis showed reasonable agreement between the predicted and observed probabilities. In the test cohort, the Brier score was lowest for HANS-RC (0.164) compared with 0.197 for HANS-Rad and 0.237 for HANS-Clin, suggesting improved overall prediction accuracy. Decision curve analysis further supported the advantage of the combined model, with HANS-RC providing consistently higher net benefit than either single-modality model as well as the “treat-all” and “treat-none” strategies across most clinically relevant threshold probabilities. Model-derived stratification remained significantly associated with OS, indicating that the model captures biologically relevant features linked to prognosis (Figure 3). Additional internal validation showed that HANS-RC retained moderate discriminatory performance under repeated cross-validation, while bootstrap optimism correction and feature-stability analyses provided complementary evidence regarding its internal robustness, although residual instability cannot be excluded (Table S5).

### 3.4. Genomic Differences Between Predicted HANS-High and HANS-Low by HANS-RC Model

**Figure 4:**
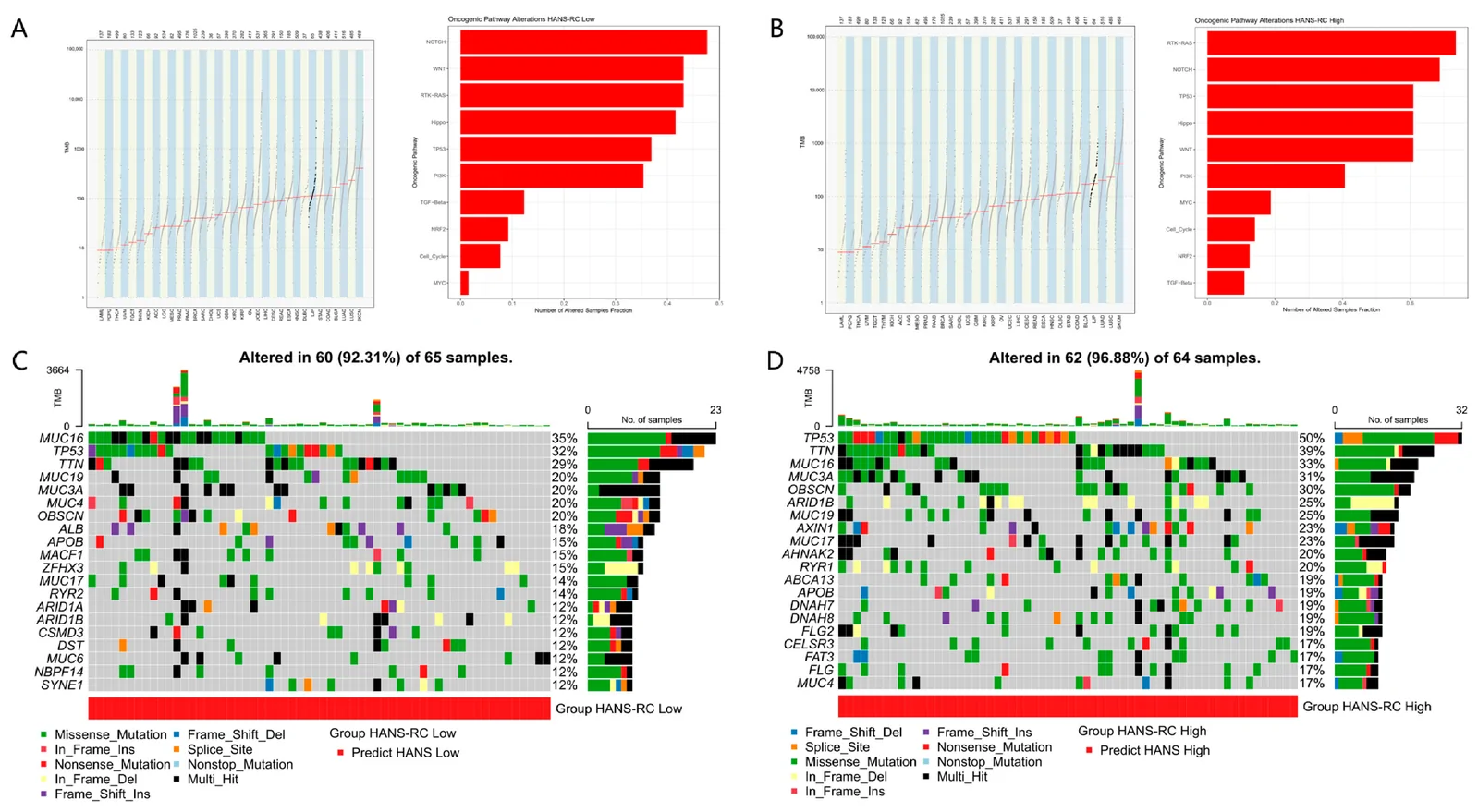
Genetic alteration profiles of HANS-RC subgroups. (**A**,**B**) TMB distribution and pathway alterations in HANS-RC low (**A**) and high (**B**) groups. Bars indicate the fraction of samples with pathway alterations. (**C**,**D**) Waterfall plots of recurrent somatic mutations. Alterations were observed in 92.3% (60/65) and 96.9% (62/64) of tumors, respectively. RTK–RAS and TP53 pathway alterations were more frequent in the high group, while the low group showed a broader distribution across pathways.

To examine whether the imaging-based stratification reflects underlying tumor biology, we compared genomic alterations between the HANS-High and HANS-Low groups by HANS-RC model. HANS-High tumors showed a higher mutational burden than HANS-Low (median TMB: 175 vs. 112 mutations per sample), with hypermutated outliers observed only in HANS-High (Figure 4B). At the gene level, HANS-High more frequently involved TP53 and large genes (TTN, MUC3A, OBSCN), together with AXIN1 and ARID1B alterations, whereas HANS-Low more often showed mutations in ARID1A, mucin family genes (MUC16, MUC4, MUC6), and ALB and ZFHX3 (Figure 4A).

Missense mutations were predominant in both groups. HANS-High showed a higher proportion of truncating events, including nonsense mutations and frameshift deletions, whereas HANS-Low showed an increase in multi-hit events and in-frame deletions.

### 3.5. Functional Characterization of Subgroup Differences

Functional enrichment analyses revealed that the two subgroups differ in key biological processes. At the pathway level, HANS-High involved a larger fraction of samples with alterations in the RTK–RAS, NOTCH, WNT, Hippo, and TP53 pathways, along with MYC-related events (Figure 4D). In contrast, HANS-Low showed greater involvement of the PI3K pathway, while NRF2 alterations affected fewer samples. TGF-β and cell cycle pathways were similar between groups (Figure 4C).

**Figure 5:**
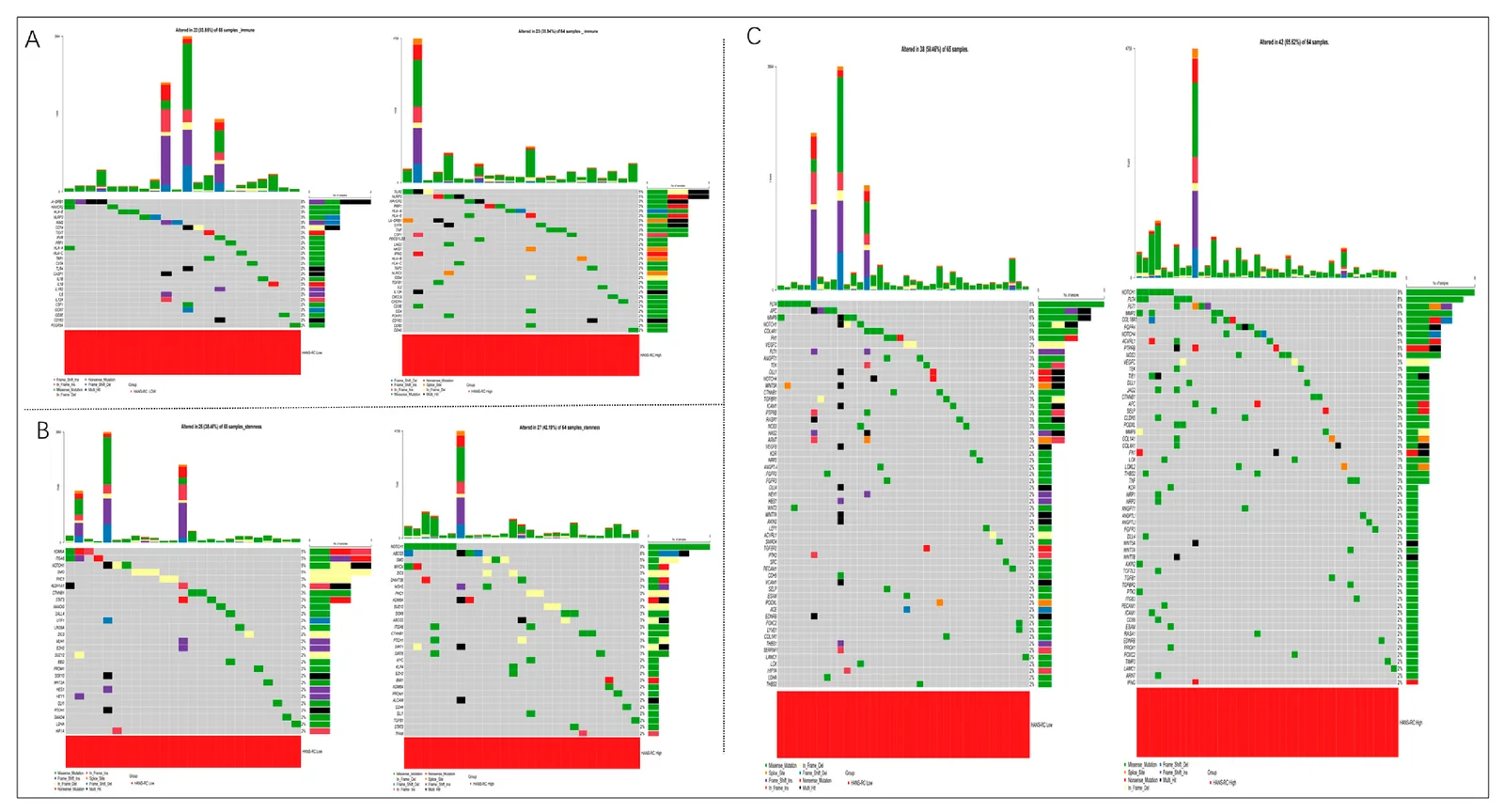
Genomic alterations across functional programs stratified by HANS-RC. (**A**–**C**) Mutation landscapes of genes related to immune activity (**A**), stemness (**B**), and angiogenesis (**C**) in the HANS-Low (**left**) and HANS-High (**right**) groups. Each panel shows the distribution and frequency of somatic alterations across samples, highlighting distinct mutational patterns between groups. These results further support that the biological relevance of HANS is associated with distinct genomic patterns in HCC.

Immune-related alterations were more frequent in HANS-High, including genes involved in antigen presentation, adaptive immune response, and checkpoint pathways, whereas HANS-Low mutations were mainly concentrated in HLA-DRB1 and inflammasome-related genes with limited adaptive immune involvement (Figure 5A). Distinct patterns were also seen in stemness-associated genes, with NOTCH1 and MYC-related alterations showing a higher observed frequency in HANS-High, while HANS-Low was characterized by pluripotency- and hypoxia-related genes (Figure 5B).

For angiogenesis, HANS-Low more frequently harbored mutations in genes such as FLT4, MMP9, and extracellular matrix-related components, while no dominant pattern was observed in HANS-High (Figure 5C).

At the global mutation level, HANS-High showed a higher proportion of C > T substitutions and more T > A events, whereas HANS-Low had a relatively higher proportion of C > G substitutions (Figure S1A). Consistent with this, the most frequently mutated genes differed between groups, with TP53 and TTN more common in HANS-High, and MUC16, ALB, and ZFHX3 more frequent in HANS-Low (Figure S3–S5).

### 3.6. HANS-RC Model Interpretation

**Figure 6:**
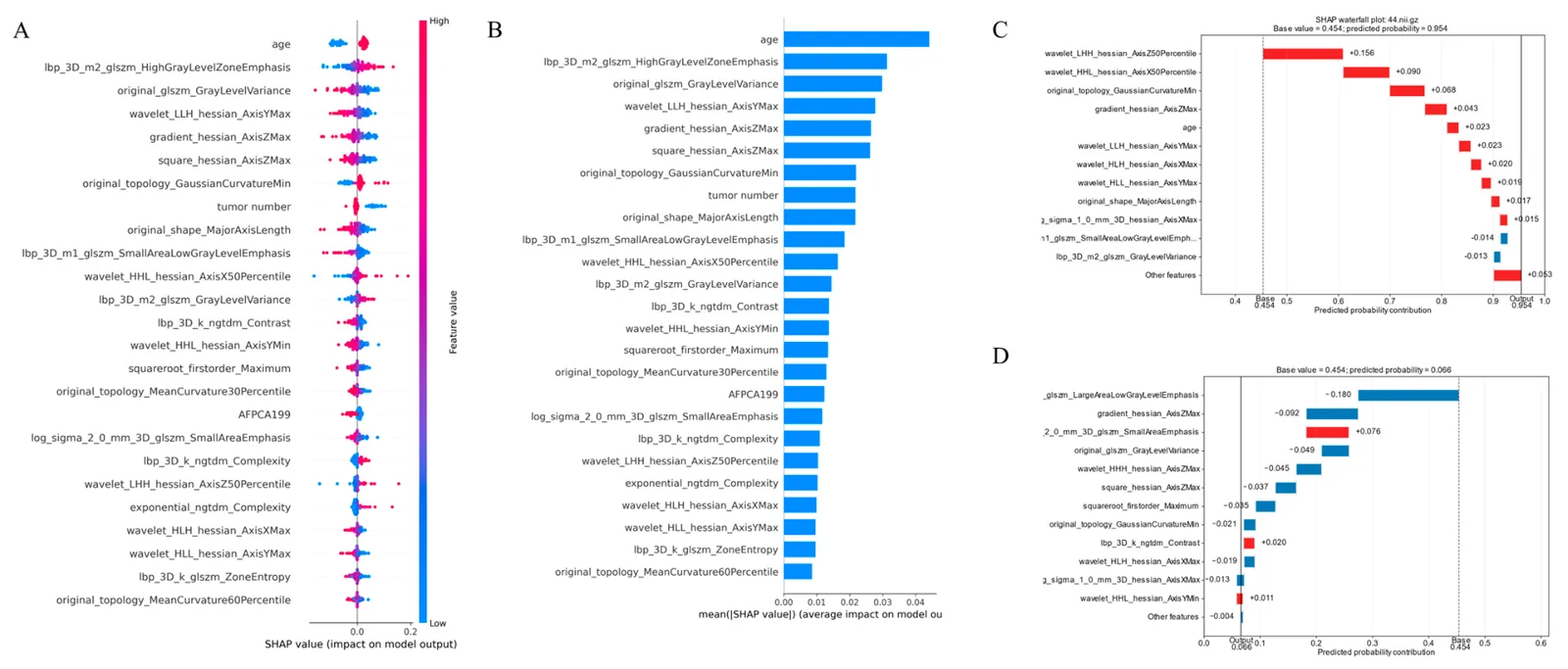
Model interpretability using SHAP. (**A**) SHAP summary plot showing the global distribution of feature effects; each point represents an individual patient, with color indicating feature value and horizontal position indicating impact on model output. (**B**) Mean absolute SHAP values ranking features by overall importance. (**C**,**D**) Representative SHAP waterfall plots illustrating feature contributions at the individual level for correctly classified cases, highlighting how radiomic and clinical variables jointly drive model predictions.

SHAP analysis was performed to interpret feature contributions to HANS-RC predictions. Global SHAP importance showed that both clinical and radiomic features contributed (Figure 6A). Age was the most influential feature, followed by radiomic features including lbp_3D_m2_glszm_HighGrayLevelZoneEmphasis, original_glszm_GrayLevelVariance, and wavelet/hessian-based features; tumor number also ranked among the top contributors.

The SHAP summary plot (Figure 6B) shows the heterogeneous and nonlinear effects across patients. Waterfall plots illustrate individualized prediction pathways. In a representative high-risk case (Figure 6C), multiple radiomic features increased the predicted probability, whereas in a low-risk case (Figure 6D), several features reduced the prediction.

## 4. Discussion

In this study, the HANS-High group showed significantly longer overall survival than the HANS-Low group (median 23 months vs. 10 months) in patients with unresectable advanced HCC receiving TACE plus antiangiogenic and ICIs. Multivariate analysis confirmed HANS as an independent protective factor, and the multi-omics prediction model built on this basis achieved satisfactory performance.

HANS-High tumors have been reported to be enriched in tumor-reactive CD39^+^CD8^+^ T cells with enhanced cytotoxic activity. TACE, however, induces both immunogenic and immunosuppressive changes [23]; single-cell data have shown reductions in CD8^+^ T cells and tumor-specific subsets following treatment, together with the expansion of TREM2^+^ immune populations [24]. Although derived from different settings, these observations suggest that treatment may reshape pre-existing antitumor immunity.

In our cohort, HANS-High patients had longer OS among patients receiving TACE-based triplet therapy, supporting a context-dependent advantage of this subgroup. Prior studies have shown that tyrosine kinase inhibitors such as donafenib or lenvatinib can increase CD8^+^ T cell infiltration, and CD8^+^ T cell abundance as well as regulatory T cell proportion have been associated with clinical outcomes [25].

Clinical variables provided additional stratification value. Among the clinical variables, concurrent elevation of AFP and CA19-9 contributed more strongly than either marker alone, consistent with reports linking CA19-9 to more aggressive tumor features [26]. Female sex is an independent protective factor for HCC incidence and progression, with a survival advantage that is age-dependent and peaks around early perimenopause [27]. Older patients have shown comparable outcomes with immune checkpoint inhibitors [28,29], suggesting that age alone should not preclude treatment.

Although HANS provides a biologically meaningful classification, its application is limited by the need for genomic sequencing and HLA typing, which are not routinely available in advanced HCC. To address this, we developed a radiomics–clinical model based on contrast-enhanced CT and routinely collected clinical variables. The predictive efficacy of radiomics in HCC treated with TACE has been widely demonstrated [30,31]. The model showed good performance in distinguishing HANS-High from HANS-Low, offering a noninvasive approach for patient stratification. By using HANS as an intermediate phenotype rather than directly predicting survival, this approach may improve interpretability and reduce the risk of overfitting.

To further support the biological basis of the model, genomic features were compared between the two subgroups. HANS-High tumors showed a higher tumor mutation burden and were characterized by a predominance of missense mutations, along with a higher frequency of truncating alterations, consistent with TP53-associated genomic instability. In contrast, HANS-Low tumors exhibited a more heterogeneous mutation pattern with dispersed alterations [32]. Immune-related differences were also evident. HANS-High tumors involved a broader spectrum of genes associated with antigen presentation and T cell function, including HLA molecules, TAP2, PRF1, and IFNG, whereas alterations in HANS-Low tumors were more restricted and tended to involve innate immune and inflammasome-related pathways. In addition, distinct tumor-intrinsic programs were observed. HANS-High tumors more frequently harbored alterations in the stemness- and proliferation-related pathways, including NOTCH and MYC signaling, while HANS-Low tumors were more frequently altered for hypoxia- and metabolism-associated genes such as HIF1A and LDHA, as well as angiogenesis-related alterations. HANS is derived from somatic mutations and predicted neoantigens; its association with mutation count is expected and does not constitute independent biological validation. The observed gene- and pathway-level patterns should therefore be interpreted as exploratory genomic characterization and require validation in independent cohorts using orthogonal transcriptomic, immunohistochemical, or spatial analyses [33].

SHAP analysis showed that HANS-RC predictions were driven by both clinical and radiomic inputs. Tumor number was the only clinical variable consistently among the top contributors. Effects were not uniform across patients. The summary plot and case-level waterfall plots illustrate this variability: radiomic features tended to accumulate positive contributions in high-risk cases, while mixed or negative contributions were more common in low-risk cases. Previous studies have linked radiomic features to tumor heterogeneity in HCC, but most have focused on direct outcome prediction. In contrast, the present study anchors imaging features to a biologically defined immunogenic subtype, providing a more mechanistically grounded framework. This approach links imaging phenotypes to immune-related tumor states rather than relying solely on statistical associations. Recent HCC studies using topological intratumoral heterogeneity and habitat-based imaging indicate that spatially resolved phenotyping may capture regional information beyond whole-tumor summary features [34,35]. Our model used whole-tumor features from a single index lesion and was not designed to identify spatial subregions. In contrast, our whole-tumor radiomics approach reflects a global imaging phenotype, and future spatially resolved analyses may provide additional insights into the intratumoral distribution of HANS-associated imaging features.

This study has several limitations. First, this retrospective single-center study had a limited sample size, precluding robust subgroup analyses, and used only internal 8:2 split-sample validation; the small test cohort may have yielded imprecise performance estimates. Second, the robustness of HANS across specific TKI and ICI regimens requires further evaluation. Third, WES-based HANS requires available biopsy tissue, which may limit generalizability and exclude some clinically diagnosed patients. Finally, whole-tumor radiomics does not explicitly resolve intratumoral spatial heterogeneity, and the WES-based genomic findings remain exploratory. Future studies incorporating spatially matched imaging-molecular profiling and orthogonal transcriptomic, metabolomic, or immunologic validation are warranted.

## 5. Conclusions

This study provides evidence that HANS-defined subgroups capture meaningful biological heterogeneity in patients with HCC treated with TACE-based triplet therapy. By integrating radiomics with genomic data, we show that imaging features are not only associated with outcomes, but also mirror underlying molecular differences, offering a non-invasive window into tumor biology. The HANS-RC model, therefore, may serve as a practical tool for stratifying patients with distinct prognostic profiles in routine clinical settings. The observed genomic divergence between HANS-High and HANS-Low further points to potential therapeutic relevance. Differences in pathway alterations and immune-related features suggest that these subgroups may respond differently to systemic treatments, raising the possibility that imaging-based phenotyping could assist in identifying patients with more favorable survival outcomes and facilitating prognostic stratification in this treatment setting. Nonetheless, these findings provide a basis for further validation and mechanistic studies. Ongoing work at our center focuses on expanding patient cohorts and exploring how this integrative approach can be translated into clinical decision-making. Ultimately, such strategies may help refine patient selection and support more individualized treatment in HCC.

## Figures and Tables

**Table 1:** Baseline characteristics of the enrolled patients with HCC.

Characteristic	Overall *N* = 129 ^1^	Low *N* = 70 ^1^	High *N* = 59 ^1^	*p*-Value
**Chronic hepatitis, n (%)**				0.185 ^2^
None	9 (7.0%)	6 (8.6%)	3 (5.1%)	
HBV	116 (90%)	63 (90%)	53 (90%)	
HCV	3 (2.3%)	0 (0%)	3 (5.1%)	
HBV + HCV	1 (0.8%)	1 (1.4%)	0 (0%)	
**BCLC,** ***N*** **(%)**				0.243 ^3^
B	31 (24%)	14 (20%)	17 (29%)	
C	98 (76%)	56 (80%)	42 (71%)	
**CNLC,** ***N*** **(%)**				0.169 ^3^
II	32 (25%)	14 (20%)	18 (31%)	
III	97 (75%)	56 (80%)	41 (69%)	
**Gender,** ***N*** **(%)**				0.084 ^3^
Female	13 (10%)	10 (14%)	3 (5.1%)	
Male	116 (90%)	60 (86%)	56 (95%)	
**Age**	54 (44–62)	50 (42–59)	56 (48–64)	0.051 ^4^
**Age_group (>50),** ***N*** **(%)**				0.015 ^3^
≤50	52 (40%)	35 (50%)	17 (29%)	
>50	77 (60%)	35 (50%)	42 (71%)	
**PS,** ***N*** **(%)**				0.421 ^3^
0	76 (59%)	39 (56%)	37 (63%)	
1	53 (41%)	31 (44%)	22 (37%)	
**Child–Pugh,** ***N*** **(%)**				0.255 ^3^
A	99 (77%)	51 (73%)	48 (81%)	
B	30 (23%)	19 (27%)	11 (19%)	
**Liver cirrhosis,** ***N*** **(%)**				0.358 ^3^
Negative	47 (36%)	23 (33%)	24 (41%)	
Positive	82 (64%)	47 (67%)	35 (59%)	
**CA199 high,** ***N*** **(%)**				0.475 ^3^
≤35	81 (63%)	42 (60%)	39 (66%)	
>35	48 (37%)	28 (40%)	20 (34%)	
**CEAs high,** ***N*** **(%)**				0.158 ^3^
≤5	111 (86%)	63 (90%)	48 (81%)	
>5	18 (14%)	7 (10%)	11 (19%)	
**CA125 high,** ***N*** **(%)**				0.177 ^3^
≤35	53 (41%)	25 (36%)	28 (47%)	
>35	76 (59%)	45 (64%)	31 (53%)	
**AFP high,** ***N*** **(%)**				0.114 ^3^
≤400	29 (22%)	12 (17%)	17 (29%)	
>400	100 (78%)	58 (83%)	42 (71%)	
**AFP CA199,** ***N*** **(%)**				0.079 ^3^
Other	93 (72%)	46 (66%)	47 (80%)	
AFP (>20), CA199 (>35)	36 (28%)	24 (34%)	12 (20%)	
**Tumor number,** ***N*** **(%)**				<0.001 ^3^
≤3	36 (28%)	11 (16%)	25 (42%)	
>3	93 (72%)	59 (84%)	34 (58%)	
**Size,** ***N*** **(%)**				0.064 ^3^
≤5	22 (17%)	8 (11%)	14 (24%)	
>5	107 (83%)	62 (89%)	45 (76%)	
**Tumor thrombus,** ***N*** **(%)**				0.107 ^3^
negative	41 (32%)	18 (26%)	23 (39%)	
positive	88 (68%)	52 (74%)	36 (61%)	
**Tumor metastasis,** ***N*** **(%)**				0.148 ^3^
negative	81 (63%)	40 (57%)	41 (69%)	
positive	48 (37%)	30 (43%)	18 (31%)	
**WBC, Mean (SD)**	7.0 (3.5)	7.1 (3.3)	6.9 (3.8)	0.834 ^4^
**Neutrophils, Mean (SD)**	4.66 (2.93)	4.74 (2.84)	4.57 (3.04)	0.757 ^4^
**Lymphocyte, Mean (SD)**	1.42 (0.65)	1.43 (0.61)	1.40 (0.69)	0.775 ^4^
**Hb, Mean (SD)**	129 (23)	127 (24)	132 (21)	0.227 ^4^
**PLT, Mean (SD)**	212 (118)	229 (137)	192 (89)	0.064 ^4^
**PT, Mean (SD)**	12.60 (1.15)	12.53 (1.17)	12.68 (1.14)	0.474 ^4^
**ALT, Mean (SD)**	51 (53)	57 (66)	44 (30)	0.160 ^4^
**AST, Mean (SD)**	84 (95)	98 (120)	66 (48)	0.047 ^4^
**ALB, Mean (SD)**	35.9 (4.7)	35.4 (5.0)	36.4 (4.2)	0.253 ^4^
**TBIL, Mean (SD)**	24 (29)	29 (38)	19 (11)	0.041 ^4^
**Cr, Mean (SD)**	79 (39)	75 (19)	84 (54)	0.217 ^4^
**Cholinesterase, Mean (SD)**	5151 (1850)	5060 (2080)	5259 (1544)	0.534 ^4^

^1^ n (%); Mean (SD). ^2^ Fisher’s exact test. ^3^ Pearson’s Chi-squared test. ^4^ Welch Two Sample *t*-test. Abbreviations: AFP, alpha-fetoprotein; ALB, albumin; ALT, alanine aminotransferase; AST, aspartate aminotransferase; BCLC, Barcelona Clinic Liver Cancer; CNLC, China Liver Cancer staging; PLT, platelet count; PT, prothrombin time; TBIL, total bilirubin.

## Data Availability

All data supporting the findings of this study have been provided as Supplementary Tables and Source Data Files. Any additional information required to reanalyze the data reported in this article is available from the lead contact upon request.

## Supplementary Materials

The following supporting information can be downloaded at: https://www.mdpi.com/article/10.3390/cancers18193218/s1, Table S1: Median follow-up time for the entire cohort (months, 95% CI); Table S2: Median follow-up time for the entire cohort (months, 95% CI); Table S3: multivariable_cox; Table S4: Univariable Cox regression and log-rank test comparing HANS-High versus HANS-Low for overall survival; Table S5. Additional internal validation and feature-selection stability of the HANS-RC model; Table S6. Spatial matching and sensitivity analyses of the HANS-RC model; Figure S1: (A–C) LASSO coefficient profiles of the HANS-Clin, HANS-Rad, and HANS-RC models, respectively. Each curve represents the coefficient trajectory of an individual feature as a function of the regularization parameter (λ). Vertical dashed lines indicate the optimal λ value selected by cross validation. (D–F) Tenfold cross validation results for the HANS-Clin, HANS-Rad, and HANS-RC models. The mean squared error (MSE) is plotted against log(λ), with error bars representing standard deviation. The dashed lines correspond to the optimal λ that minimizes the cross validated error. (G–I) Selected features and their corresponding coefficients in the final models. (G) Clinical variables retained in the HANS-Clin model. (H) Radiomic features selected in the HANS-Rad model. (I) Combined clinical and radiomic features included in the HANS-RC model, with coefficients indicating their relative contribution to the model; Figure S2: Kaplan–Meier curves of overall survival stratified by true HANS status (A,B) and by HANS status predicted by the HANS-RC model (C,D) in the training (A,C) and validation (B,D) cohorts. In the training cohort, both true and model predicted HANS-High groups showed better survival than their respective low groups, whereas no significant separation was observed in the validation cohort. Notably, in the combined cohort, patients classified as HANS-High by the HANS-RC model demonstrated a clear survival advantage. Tick marks indicate censored observations, and numbers at risk are shown below each plot; Figure S3: (A) Distribution of variant classifications, showing that missense mutations represent the predominant alteration type, followed by frameshift insertions and deletions, in-frame mutations, and nonsense mutations. (B) Distribution of variant types, indicating that single nucleotide polymorphisms (SNPs) constitute the majority of genomic alterations, with fewer insertions (INS) and deletions (DEL). (C) Single nucleotide variant (SNV) classes, demonstrating that C > T transitions are the most frequent substitution type, followed by T > A, C > A, and other substitution patterns. (D) Number of variants per sample, illustrating the overall mutation burden distribution across patients in the HANS-Low group. (E) Summary of variant classifications across samples, highlighting the relative contribution of different mutation types within individual tumors. (F) Top 10 mutated genes in the HANS-Low group, with mutation frequencies indicated, showing that genes such as TTN, MUC16, TP53, and ALB are among the most frequently altered. Overall, the HANS-Low group exhibits a mutation spectrum characterized by predominant missense mutations and SNPs, with recurrent alterations in canonical HCC-associated genes; Figure S4: (A) Distribution of variant classifications, showing that missense mutations are the predominant alteration type, followed by in-frame deletions, frameshift insertions/deletions, and nonsense mutations. (B) Distribution of variant types, indicating that single nucleotide polymorphisms (SNPs) account for the majority of genomic alterations, with a smaller proportion of insertions (INS) and deletions (DEL). (C) Single nucleotide variant (SNV) classes, demonstrating that C > T transitions are the most frequent substitution type, followed by T > A, C > A, and other substitution patterns. (D) Number of variants per sample, illustrating the distribution of tumor mutation burden across patients in the HANS-High group. (E) Summary of variant classifications across samples, highlighting the contribution of different mutation types within individual tumors. (F) Top 10 mutated genes in the HANS-High group, with mutation frequencies indicated, showing that genes such as TTN, TP53, MUC16, MUC3A, ARID1B, and AXIN1 are frequently altered. Figure S5. Gene Ontology biological process enrichment analysis of genes in the HANS-RC High and HANS-RC Low groups. (A) Significantly enriched Gene Ontology biological process (GO-BP) terms in the HANS-High group. (B) Significantly enriched GO-BP terms in the HANS-Low group. Bar length represents the number of genes associated with each GO-BP term (Count), and bar color indicates the multiple-testing-adjusted *p* value (P.adjust). Only significantly enriched terms with P.adjust < 0.05 are shown.(C) Venn diagram showing the overlap of significantly enriched GO-BP terms between the HANS-High and HANS-Low groups. A total of 420 and 192 GO-BP terms were unique to the HANS-High and HANS-Low groups, respectively, whereas 324 terms were shared between the two groups.

## Abbreviations

AFP, alpha-fetoprotein; ALB, albumin; ALT, alanine aminotransferase; AST, aspartate aminotransferase; AUC, area under the receiver operating characteristic curve; BCLC, Barcelona Clinic Liver Cancer; CA19-9, carbohydrate antigen 19-9; CEA, carcinoembryonic antigen; CI, confidence interval; CNLC, China Liver Cancer staging; CT, computed tomography; DCA, decision curve analysis; DICOM, Digital Imaging and Communications in Medicine; DNA, deoxyribonucleic acid; ECOG, Eastern Cooperative Oncology Group; HANS, high-affinity neoantigen score; HCC, hepatocellular carcinoma; HBV, hepatitis B virus; HCV, hepatitis C virus; HLA, human leukocyte antigen; HR, hazard ratio; HU, Hounsfield unit; IC_50_, half-maximal inhibitory concentration; ICC, intraclass correlation coefficient; ICI, immune checkpoint inhibitor; IQR, interquartile range; ITK-SNAP, Insight Segmentation and Registration Toolkit-SNAP; KNN, k-nearest neighbors; LASSO, least absolute shrinkage and selection operator; LightGBM, Light Gradient Boosting Machine; LR, logistic regression; MHC, major histocompatibility complex; MLP, multilayer perceptron; MRI, magnetic resonance imaging; OS, overall survival; PACS, picture archiving and communication system; PD-1, programmed cell death protein 1; PD-L1, programmed death-ligand 1; PLT, platelet count; PS, performance status; PVTT, portal vein tumor thrombus; RF, random forest; ROC, receiver operating characteristic; SD, standard deviation; SHAP, Shapley Additive Explanations; SVM, support vector machine; TACE, transarterial chemoembolization; TBIL, total bilirubin; TIL, tumor-infiltrating lymphocyte; TIME, tumor immune microenvironment; TKI, tyrosine kinase inhibitor; TMB, tumor mutational burden; TNM, tumor-node-metastasis; WBC, white blood cell count; WES, whole-exome sequencing; XGBoost.
