# Supplementary material for: Interpretable CT-Based Radiomics for Prediction of High-Affinity Neoantigens Associated Immunogenic States in Advanced Hepatocellular Carcinoma Treated with TACE Plus Targeted Therapy and Immunotherapy
Source: Cancers (Basel). 2026 Oct 6;18(19):3218. doi: 10.3390/cancers18193218 (PMC13664292; doi:10.3390/cancers18193218)
Supplement: Supplementary file 1 [file cancers-18-03218-s001.zip › Supplementary Materials.pdf]

Supplementary Materials

CT Scanning Protocol and Image Acquisition

Per our institutional protocol, scans were acquired using a 320-slice CT scanner (Aquilion One; Toshiba Medical Systems, Tokyo, Japan). Images were obtained at 37 s (arterial phase) and 65 s (portal venous phase) after injection of the contrast agent (Ultravist 300, Schering, Berlin, Germany) at a dose of 1.5 mL/kg and a flow rate of 3–4 mL/s. Scanning parameters were: tube voltage 120 kV, tube current 250 mA. Reconstruction used a slice thickness of 1 mm, an interslice gap of 0.8 mm, and a 512 × 512 matrix. Baseline conventional imaging features of the tumors were assessed by reviewing both non-contrast and biphasic contrast-enhanced CT images.

Treatment Regimen

Systemic therapy was administered according to the standard dosing schedules of the respective agents. Pembrolizumab was administered intravenously at 200 mg every 3 weeks or 400 mg every 6 weeks. Sorafenib was given orally at an initial dose of 400 mg twice daily, whereas lenvatinib was administered orally once daily at 8 mg for patients weighing <60 kg and 12 mg for those weighing ≥60 kg. For patients receiving atezolizumab plus bevacizumab (T+A), atezolizumab was administered intravenously at 1,200 mg together with bevacizumab at 15 mg/kg every 3 weeks. Treatment was continued until disease progression, unacceptable toxicity, loss of clinical benefit, or other clinically indicated discontinuation, with temporary interruption or dose modification permitted according to adverse events, liver function, and individual tolerability.

Table S1 Median follow-up time for the entire cohort (months, 95% CI).

	(95%CI)
The median follow-up time	38.0 (32.0–49.0)

Table S2 The median overall survival.

Group	Median OS(months)
HANS low	10.0 (8.0–16.0)
HANS high	23.0 (12.0–32.0)

Table S3 multivariable_cox.

**Variable**[1]	***N***	**HR**[1]	**95% CI**[1]	***p*-value**[1]
TMB	129			0.935
Low		—	—	
High		1.02	0.65, 1.60	
HANS	129			**0.026**
Low		—	—	
High		0.60	0.39, 0.95	
PS	129			**<0.001**
0		—	—	
1		3.01	1.95, 4.64	
Child–Pugh	129			0.599
A		—	—	
B		1.15	0.69, 1.91	
Tumor number	129			**0.016**
≤3		—	—	
>3		1.89	1.11, 3.23	
Tumormetastasis	129			**0.008**
negative		—	—	
positive		1.78	1.17, 2.72	
size	129			**0.034**
≤5		—	—	
>5		1.88	1.01, 3.50	
tumorthrombus	129			0.643
without		—	—	
with		0.89	0.55, 1.45	

Variable[1]	*N*	HR[1]	95% CI[1]	*p*-value[1]

Abbreviations: CI = Confidence Interval, HR = Hazard Ratio

Table S4 Univariable Cox regression and log-rank test comparing HANS-high versus HANS-low for overall survival.

Group	HR (95%CI)	Cox *P* 值	Log-rank *P* 值
HANS high vs HANS low	0.51 (0.34–0.77)	0.001	<0.001

Median OS (A), median follow-up (B), and univariable Cox/log-rank results (C) for HANS-low vs. HANS-high groups.

Radiomic Feature Extraction and HANS-Rad Construction

Radiomic features extracted from arterial-phase CT totaled 2,346. After Z-score normalization and Pearson correlation filtering (r>0.9), 578 features remained and entered LASSO regression, which selected 41 features to construct the Rad-score. These were predominantly wavelet-, LBP-, and exponential-transform-based texture features (HANS-Rad, Figure S1).

The clinical model (HANS-Clin) started with 29 variables; following the same normalization and correlation filtering, 27 features underwent LASSO, yielding 13 retained features: HBV, sex, age, cirrhosis, elevated CEA, elevated AFP, combined AFP/CA19-9 elevation, tumor number, tumor size, tumor thrombus, distant metastasis, platelet count, and prothrombin time. The radiomic model used the 40 radiomic features to predict HANS status (Figure S1).

For the combined model(HANS-RC), the four highest-weight clinical features from LASSO (sex, age, tumor number, and AFP/CA19-9 elevation) were merged with the 40 radiomic features (44 total). A second LASSO selected 36 non-zero coefficients, comprising all four clinical and 32 radiomic features, which were used to build the combined model (Figure S1).

Additional internal validation

To further evaluate the internal robustness of HANS-RC, bootstrap optimism correction was performed using 1,000 bootstrap resamples in the development cohort. This analysis was conditional on the fixed 36-feature set and final MLP configuration. For

each bootstrap sample, the model was refitted and evaluated in both the bootstrap sample and the original development cohort, and the mean difference in AUC was used to estimate optimism. The optimism-corrected AUC was calculated by subtracting the mean optimism from the apparent AUC. The Brier score was corrected using the same principle.

In addition, stratified 5-fold cross-validation was repeated 10 times to evaluate the reproducibility of model discrimination. Feature-selection stability was further assessed using 300 stratified resampling iterations, in each of which 80% of the cohort was sampled without replacement. Stability was summarized by the number and proportion of retained features, the mean Jaccard index, and the Nogueira stability index.

Table S5. Additional internal validation and feature-selection stability of the HANS-RC model.

Analysis	Metric	Result
Bootstrap optimism correction	Bootstrap resamples	1,000
	Apparent AUC	0.980
	Mean optimism	0.074
	Optimism-corrected AUC	0.906
	Empirical interval	0.856–0.943
	Apparent Brier score	0.080
	Optimism-corrected Brier score	0.147
Repeated cross-validation	Validation scheme	5-fold × 10 repeats
	Mean AUC	0.762
	Conditional 95% interval	0.684–0.836
Feature-selection stability	Resampling iterations	300
	Sampling fraction	80%
	Mean retained features	27.6/36
	Mean retention proportion	76.7%
	Range of retained features	16–36
	Mean Jaccard index	0.721

The bootstrap optimism analysis was conditional on the fixed 36-feature set and final MLP configuration and therefore does not account for uncertainty arising from the complete feature- and classifier-selection process.

Spatial matching sensitivity analysis

Spatial correspondence between the lesion segmented on arterial-phase CT and the lesion sampled for whole-exome sequencing (WES) was retrospectively reviewed for all 129 patients using anatomical location, liver segment, lesion size, and the corresponding sampling, operative, and pathological records. Only unequivocally concordant lesions were classified as spatially matched; uncertain or definitively unmatched cases were classified as not confirmed. Spatial matching was confirmed in 114 of 129 patients (88.4%), including 91 of 103 patients in the development cohort and 23 of 26 patients in the test cohort.

Two sensitivity analyses were performed. First, the original HANS-RC model and its predicted probabilities were retained without refitting, and discrimination was recalculated after restricting the independent test cohort to the 23 patients with confirmed spatial matching. No additional feature selection, model refitting, hyperparameter optimization, or classifier reselection was performed. AUCs and corresponding 95% confidence intervals were estimated using the DeLong method.

Table S6. Spatial matching and sensitivity analyses of the HANS-RC model.

Group	Result
Confirmed spatial matching, overall	114/129 (88.4%)
Not confirmed (uncertain or unmatched)	15/129 (11.6%)
Confirmed matching, development cohort	91/103 (88.3%)
Confirmed matching, test cohort	23/26 (88.5%)
Frozen HANS-RC model, matched test cohort	AUC 0.854 (95% CI 0.669–1.000)
Frozen HANS-RC model, full test cohort	AUC 0.869
ΔAUC, matched vs full test cohort	−0.015
Repeated stratified 5-fold cross-validation ×10, spatially matched evaluation	AUC 0.758 (95% CI 0.676–0.834)
Locked-model out-of-fold analysis, full cohort	AUC 0.762
ΔAUC, matched vs full cohort	−0.004

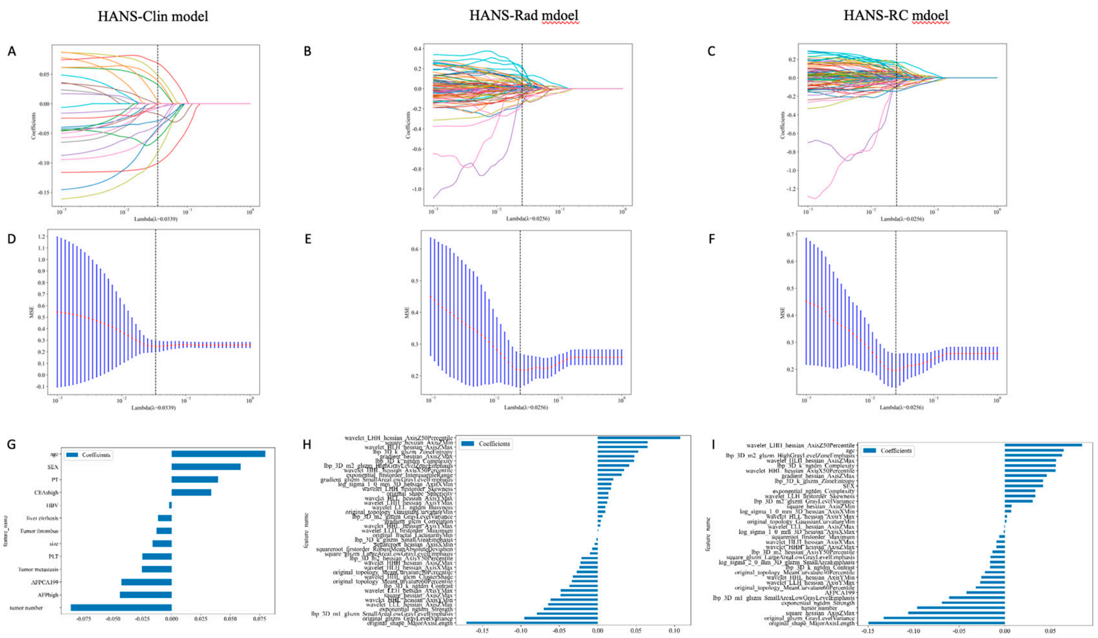

Figure S1. (A–C) LASSO coefficient profiles of the HANS-Clin, HANS-Rad, and HANS-RC models, respectively. Each curve represents the coefficient trajectory of an individual feature as a function of the regularization parameter (λ). Vertical dashed lines indicate the optimal λ value selected by cross-validation. (D–F) Ten-fold cross-validation results for the HANS-Clin, HANS-Rad, and HANS-RC models. The mean squared error (MSE) is plotted against log(λ), with error bars representing standard deviation. The dashed lines correspond to the optimal λ that minimizes the cross-validated error. (G–I) Selected features and their corresponding coefficients in the final models. (G) Clinical variables retained in the HANS-Clin model. (H) Radiomic features selected in the HANS-Rad model. (I) Combined clinical and radiomic features included in the HANS-RC model, with coefficients indicating their relative contribution to the model.

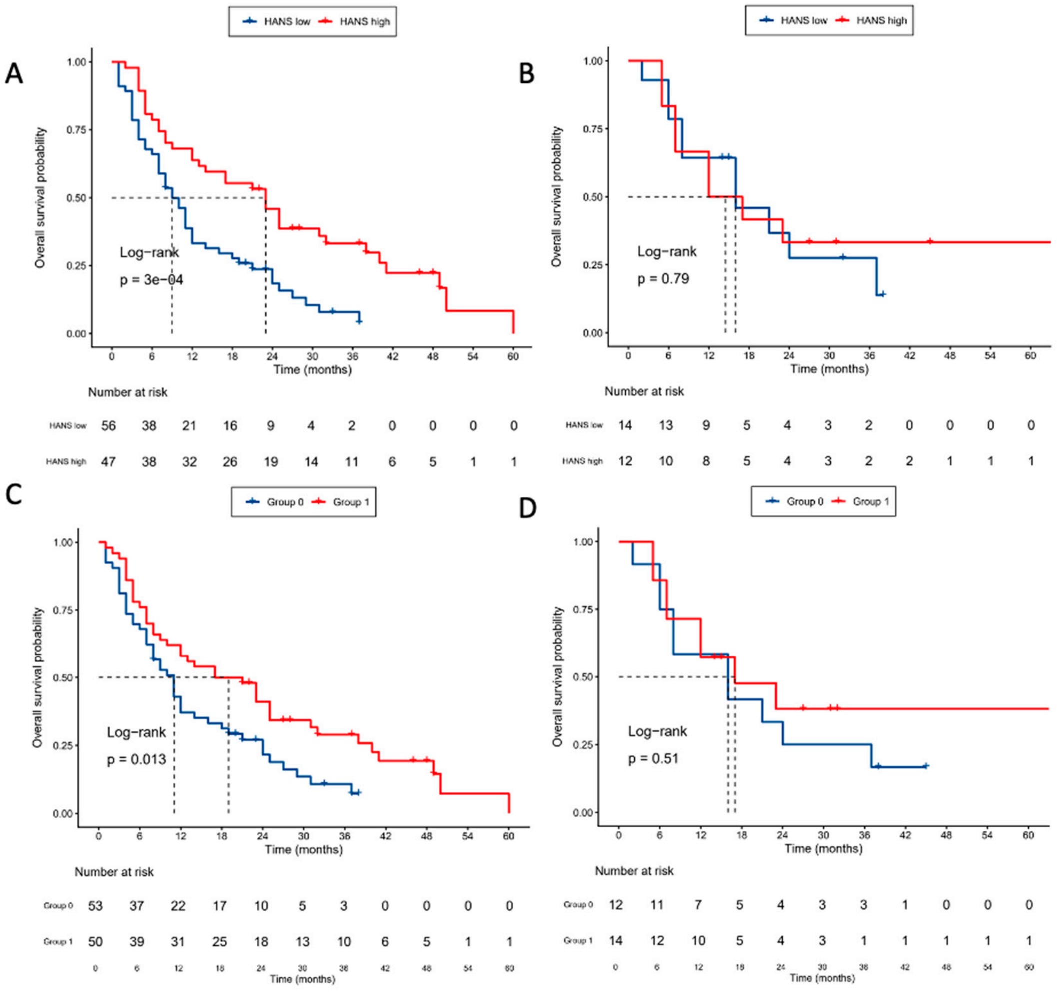

Figure S2. Kaplan–Meier curves of overall survival stratified by true HANS status (A–B) and by HANS status predicted by the HANS-RC model (C–D) in the training (A, C) and validation (B, D) cohorts. In the training cohort, both true and model-predicted HANS-high groups showed better survival than their respective low groups, whereas no significant separation was observed in the validation cohort. Notably, in the combined cohort, patients classified as HANS-high by the HANS-RC model demonstrated a clear survival advantage. Tick marks indicate censored observations, and numbers at risk are shown below each plot.

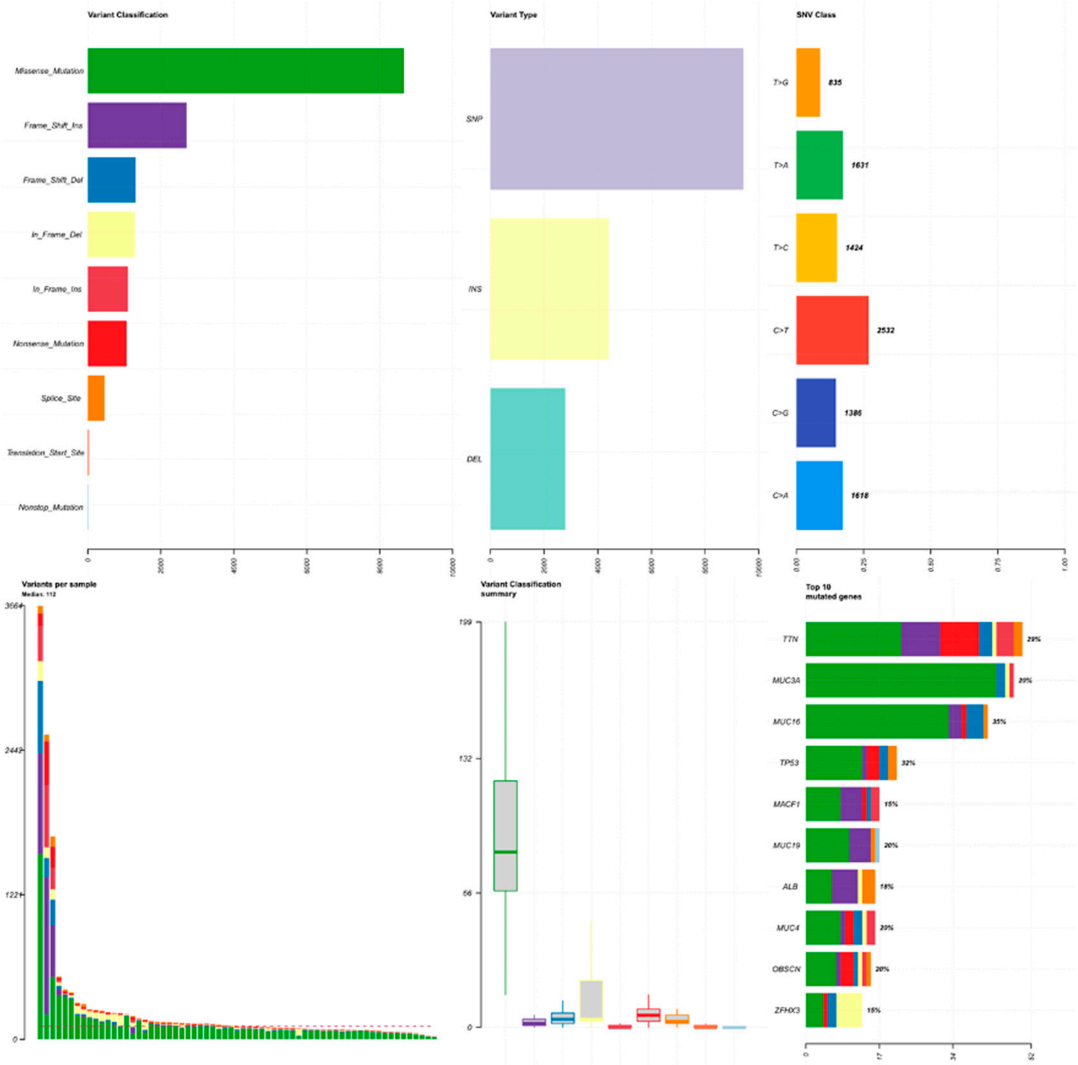

Figure S3. (A) Distribution of variant classifications, showing that missense mutations represent the predominant alteration type, followed by frameshift insertions and deletions, in-frame mutations, and nonsense mutations. (B) Distribution of variant types, indicating that single nucleotide polymorphisms (SNPs) constitute the majority of genomic alterations, with fewer insertions (INS) and deletions (DEL). (C) Single nucleotide variant (SNV) classes, demonstrating that C>T transitions are the most frequent substitution type, followed by T>A, C>A, and other substitution patterns. (D) Number of variants per sample, illustrating the overall mutation burden distribution across patients in the HANS-Low group. (E) Summary of variant classifications across samples, highlighting the relative contribution of different mutation types within individual tumors. (F) Top 10 mutated genes in the HANS-Low group, with mutation frequencies indicated, showing that genes such as *TTN*, *MUC16*, *TP53*, and *ALB* are among the most frequently altered. Overall, the HANS-Low group exhibits a mutation spectrum characterized by predominant missense mutations and SNPs, with recurrent alterations in canonical HCC-associated genes.

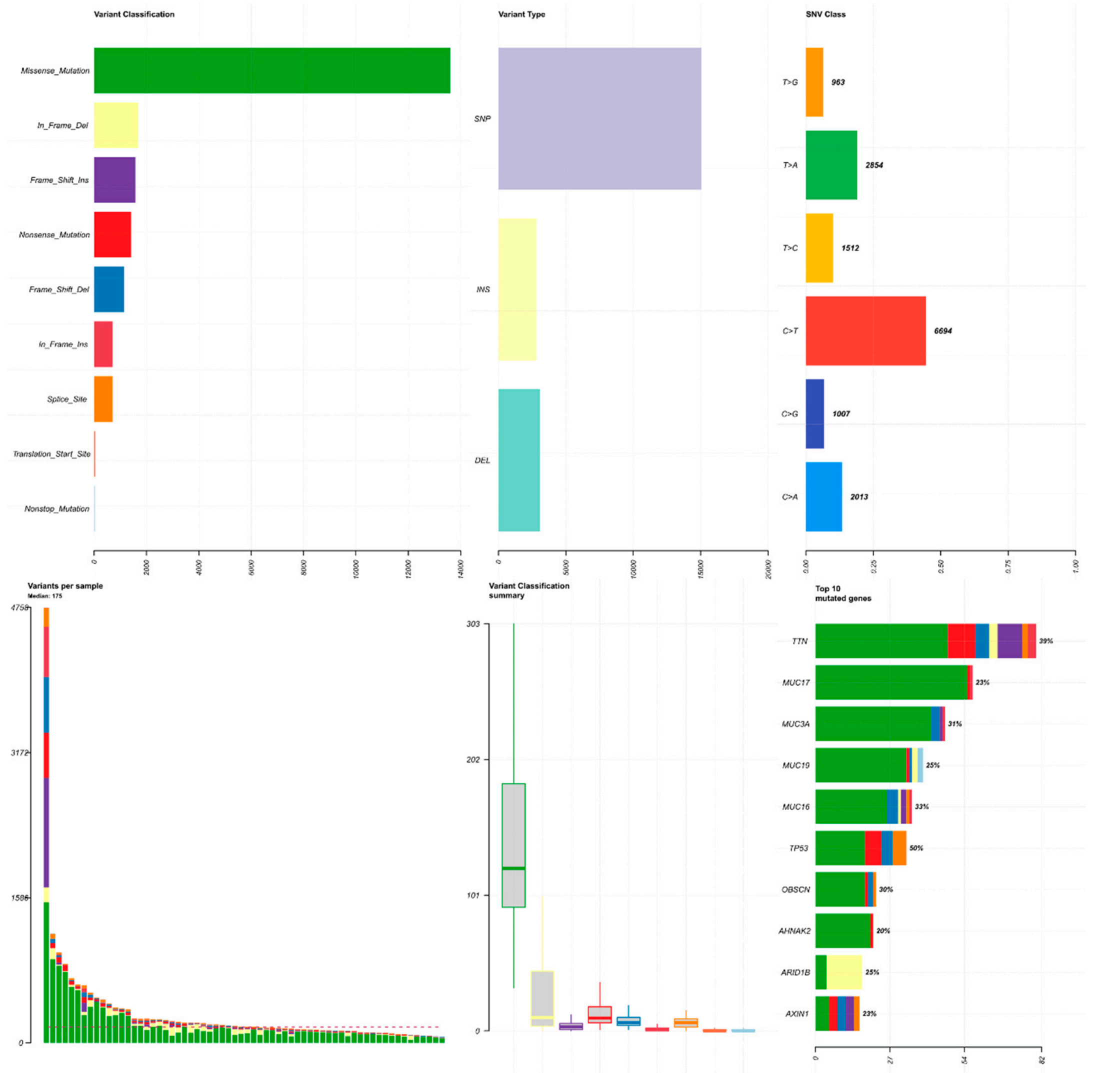

Figure S4. (A) Distribution of variant classifications, showing that missense mutations are the predominant alteration type, followed by in-frame deletions, frameshift insertions/deletions, and nonsense mutations. (B) Distribution of variant types, indicating that single nucleotide polymorphisms (SNPs) account for the majority of genomic alterations, with a smaller proportion of insertions (INS) and deletions (DEL). (C) Single nucleotide variant (SNV) classes, demonstrating that C>T transitions are the most frequent substitution type, followed by T>A, C>A, and other substitution patterns. (D) Number of variants per sample, illustrating the distribution of tumor mutation burden across patients in the HANS-High group. (E) Summary of variant classifications across samples, highlighting the contribution of different mutation types within individual tumors. (F) Top 10 mutated genes in the HANS-High group, with mutation frequencies indicated, showing that genes such as TTN, TP53, MUC16, MUC3A, ARID1B, and AXIN1 are frequently altered.

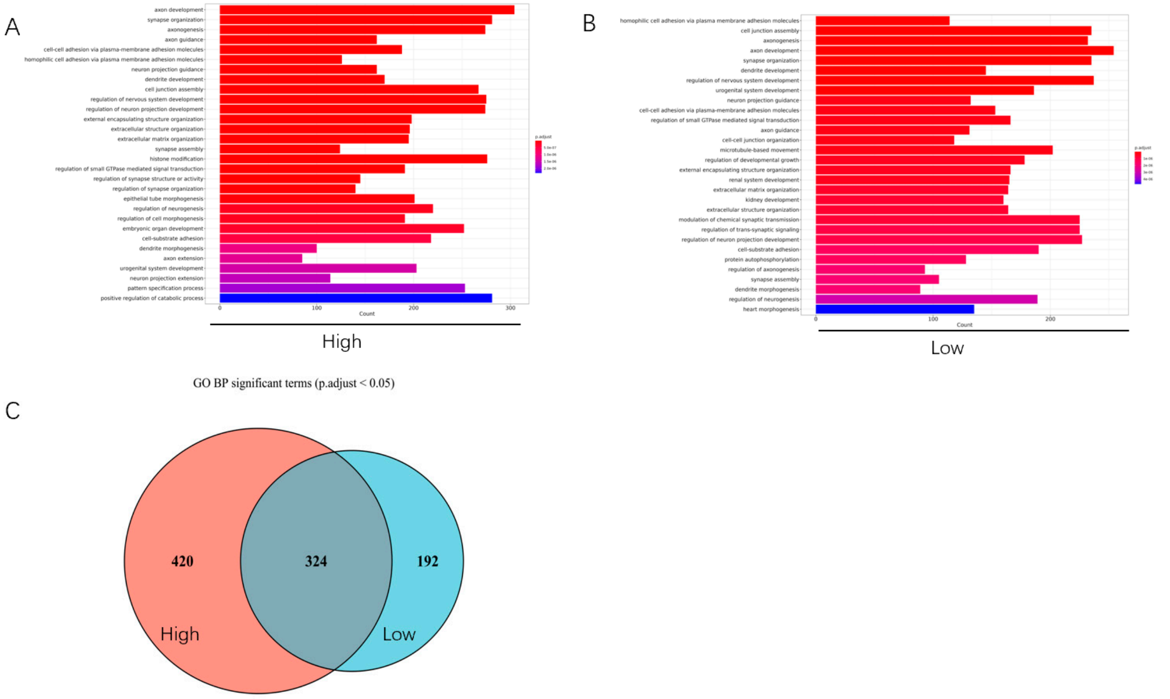

Figure S5. Gene Ontology biological process enrichment analysis of genes in the HANS-RC High and HANS-RC Low groups. (A) Significantly enriched Gene Ontology biological process (GO-BP) terms in the HANS-High group. (B) Significantly enriched GO-BP terms in the HANS-Low group. Bar length represents the number of genes associated with each GO-BP term (Count), and bar color indicates the multiple-testing-adjusted p value (*P*.adjust). Only significantly enriched terms with *P*.adjust < 0.05 are shown. (C) Venn diagram showing the overlap of significantly enriched GO-BP terms between the HANS-High and HANS-Low groups. A total of 420 and 192 GO-BP terms were unique to the HANS-High and HANS-Low groups, respectively, whereas 324 terms were shared between the two groups.
